# Too Depleted to Turn In: The Relevance of End-of-the-Day Resource Depletion for Reducing Bedtime Procrastination

**DOI:** 10.3389/fpsyg.2018.00252

**Published:** 2018-03-14

**Authors:** Bart A. Kamphorst, Sanne Nauts, Denise T. D. De Ridder, Joel H. Anderson

**Affiliations:** ^1^Department of Computer Science, Vrije Universiteit Amsterdam, Amsterdam, Netherlands; ^2^Self-Regulation Lab, Department of Social, Health and Organizational Psychology, Utrecht University, Utrecht, Netherlands; ^3^Department of Philosophy and Religious Studies, Utrecht University, Utrecht, Netherlands

**Keywords:** bedtime procrastination, behavior change, e-coaching systems, self-regulation, sleep

## Abstract

*Bedtime procrastination* is an important predictor of sleep insufficiency in the general population (Kroese et al., 2014b), but little is known about the determinants of this self-undermining behavior. As the phenomenon has been conceptualized in the literature as a form of self-regulation failure (Kroese et al., 2014a), we hypothesized that people’s self-regulatory resources in the evening would be predictive of going to bed later than they intended. Specifically, we examined whether the cumulative effect of resisting desires, a measure of self-regulatory resource depletion (Hofmann et al., 2012b), relates to bedtime procrastination. Participants (*N* = 218) reported how many desires they had tried to resist during the previous day and the extent of their bedtime procrastination. Results show that people who attempted to resist more desires were more likely to engage in bedtime procrastination, suggesting that people may be less likely to stick to their intended bedtime after a particularly taxing day. Implications for intervention strategies are discussed.

## Introduction

Since the 1940s, average sleep times have declined sharply, with 40% of Americans now sleeping less than 6 hours per night (Gallup, 2013). Sleep deprivation has serious negative consequences for people’s health and well-being: it causes hormonal and immunological disruptions that put people at risk for developing diabetes, obesity, cancer, cardiovascular disease, chronic infections, and neuropsychiatric diseases (Strine and Chapman, 2005; Irwin, 2015). Sleep deprivation reduces health-related quality of life (Paiva et al., 2015) and contributes to increased mortality (Gallicchio and Kalesan, 2009). Sleep researchers have long focused on identifying medical causes for sleep insufficiency (e.g., sleep apnea, insomnia), but given that approximately 90% of the population does not suffer from a sleep disorder (Ram et al., 2010), researchers have reason to turn their attention to *behavioral* explanations for insufficient sleep.

One behavioral line of explanation centers on understanding the familiar yet under-studied phenomenon of *bedtime procrastination*, which has been defined as “needlessly and voluntarily delaying going to bed, despite foreseeably being worse off as a result” (Kroese et al., 2016). An example of this behavior would be watching “just one more” episode of one’s favorite Netflix show despite having intended to go to bed at that hour, realizing that the delay will leave one feeling tired and irritable the next day, and having a considered preference for being well-rested the next day over having watched an additional episode. Bedtime procrastination has been shown to be related to trait self-control (Kroese et al., 2014b) and has therefore been conceptualized as a form of self-regulatory failure (Hagger, 2010; Loft and Cameron, 2013, 2014; Kroese et al., 2014a,b; Todd and Mullan, 2014). In line with this idea, we expect that people’s success in going to bed at their intended bedtime is also related to how much self-control people can muster in the critical moments of deciding whether to go to bed or to procrastinate.

In the evening, people increasingly need to rely on self-control to turn off their devices (televisions, smartphones, PlayStations, etc.) so they can turn in for a good night’s sleep. Unfortunately, people’s self-control is generally at a low point in the evening (Baumeister, 2002). After a long day at the office, people might not be able or willing to expend the self-regulatory resources needed to get oneself to bed, especially if they already expended substantial resources resisting desires throughout the day (e.g., the desire to have that delicious-looking chocolate pie for lunch; the desire to walk out of a boring meeting; the desire to watch YouTube clips instead of working on a finance report, etc. (cf. Hofmann et al., 2012a,b). As a result, we expect that people will have more difficulty going to bed at their intended bedtime if they have fewer resources available after a taxing day.

In formulating our hypothesis in terms of the depletion of self-regulatory resources, we wish neither to ignore alternative explanations nor to downplay the objections that have been raised against a “willpower muscle” explanation of self-regulation failure. Important objections to the strength model of self-control have been raised (e.g., Job et al., 2010; Carter and McCullough, 2014; Inzlicht et al., 2014). Moreover, as we discuss more fully in Section “Discussion,” self-licensing (Witt Huberts et al., 2012), a desire for mood repair (Sirois and Pychyl, 2013), and effort-allocation choices (Inzlicht and Schmeichel, 2012) may all contribute to self-regulation failure. For these reasons, our more modest aim here is to explore potential correlations between bedtime procrastination and the conditions associated with the depletion of self-regulatory resources (in particular, the number of attempts to resist desires).

If it does turn out to be the case that intention-subverting temptations are more difficult to resist as a result of being more depleted, then this opens up new avenues for predicting when bedtime procrastination is likely to occur. This could have further implications, in turn, for the type of intervention strategies, such as the use of “e-coaching systems” (Kamphorst, 2017), that would be suitable for helping people in such situations to go to bed on time, since simply “buckling down” and exerting more self-control is unlikely to succeed. Rather, people in those kinds of situations are more likely to benefit from being coached in setting up appropriate, pre-determined action plans (*implementation intentions*; Gollwitzer, 1999) that they can follow semi-automatically when they are nudged by a specific environmental cue at the appropriate moment^1^.

In the present research, we examined whether people who attempted to resist many desires (e.g., the desire to smoke, drink coffee, spend time on leisurely activities, or eat something unhealthy) during the day are indeed more likely to engage in a delay of their bedtime that was, by their own lights, unwarranted. In approaching the depletion of self-regulatory resources in this way, we are drawing on an approach developed by Hofmann et al. (2012b) in which participants had to indicate, throughout the day, whether they experienced certain desires (e.g., the desire for coffee, tobacco, leisure, and eating) and whether they had attempted to resist that desire. In the present study, participants reported retrospectively the number of times they attempted to resist a desire. Based on these results, we calculated a depletion index, as a function of the number of *resist attempts* (here, we borrow the terminology from Hofmann et al., 2012b) in which more recent resist attempts received more weight than earlier resist attempts. In previous research, this depletion index predicted the likelihood of self-regulatory success (Hofmann et al., 2012b). Put differently, people have previously been shown to be more likely to give in to temptation after they had attempted to resist many desires. For example, they were more likely to eat a tempting piece of chocolate cake for lunch after having spent all morning trying to resist desires to smoke, drink coffee, or watch funny YouTube videos instead of working on a finance report. In a similar vein, we expected that participants would be more likely to engage in bedtime procrastination if they had attempted to resist many desires on a given day.

## Materials and Methods

### Participants

The study involved 245 individuals who participated through Amazon’s Mechanical Turk (MTurk) in exchange for $1. The median time to complete the survey was about 8 min, which brings the wage to approximately $7,50 per hour. The MTurk reputation of all the participants was above 97%, which is an indication that these participants provide high-quality data (Peer et al., 2014). Twenty-seven participants (11%) were removed from the sample because they had been diagnosed with a sleep disorder (17 participants) or worked night shifts (12 participants; categories are partly overlapping)^2^. The remaining sample consisted of 218 participants (119 men) with an average age of 35 (range: 18–75 years). All participants gave their written informed consent.

### Materials

#### Bedtime Procrastination Scale

To measure bedtime procrastination, we created a state version of the bedtime procrastination scale (BPS; Kroese et al., 2014b). Whereas the BPS asks people to report whether they *generally* engage in bedtime procrastination (examples include: “I go to bed later than intended”; “Often, I am still doing other things when it is time to go to bed”), the state version asks participants to report whether they engaged in bedtime procrastination in a specific time period – in this case, yesterday (example items: “*Yesterday*, I went to bed later than I had intended”; “*Yesterday*, I was still doing other things when it was time to go to bed”). The scale comprised nine items that had high inter-item reliability (α = 0.94). Items were measured on a 7-point scale (*completely false–completely true*); on average, participants’ BPS-score was 3.37 (*SD* = 1.71).

#### Duration of Bedtime Procrastination

As a second operationalization of bedtime procrastination, we asked participants to indicate whether they had engaged in bedtime procrastination the night before and, if they had, for how long (in minutes). Bedtime procrastination was defined as “going to bed later than you would have wanted to, even though you could have gone to bed earlier.” On average, the duration of participants’ bedtime procrastination was 22.05 min (*SD* = 44.47). Sixty-seven percent of participants indicated they had not engaged in bedtime procrastination on the previous night: for them, *Duration of Bedtime Procrastination* was 0 min. The *BPS* and *Duration of Bedtime Procrastination* had a correlation of Spearman’s *r* = 0.64 (*p* < 0.001).

### Procedure

After having given informed consent, participants were first instructed to think back to the previous day. We divided the day into six timeslots of 3 hours each (e.g., *Timeslot 1* was *between getting up and 9 am* and *Timeslot 2* was *between 9 am and noon*) and asked participants to briefly write down what they did *yesterday* and how they felt during each of these timeslots. In including these questions, our study differs from the experience sampling used in previous research (Hofmann et al., 2012a,b), in which participants were asked at various points throughout the day to report how many desires they had attempted to resist in the previous timeslot. As we used a retrospective version of the Hofmann et al. (2012b) method, we included these extra questions to help people recall the events of the previous day in order to facilitate their recall of the desires they experienced. After writing down what they did during each timeslot, participants were subsequently asked to indicate whether, during each timeslot, they had experienced a desire for things like *coffee*, *leisure*, and *tobacco* (reported in terms of the *desire index;* see **Table 1** for a full list of items; participants could select more than one item). Third, they were asked whether they had tried to resist those desires. For example, if participants indicated having experienced a desire for *coffee*, *leisure*, and *tobacco* between getting up and 9 am, participants would be asked whether they had tried to resist this desire for *coffee* (yes/no), *leisure* (yes/no), and *tobacco* (yes/no) between getting up and 9 am. Fourth, participants were asked to complete, in retrospect, a state version of the BPS, which contained questions about their bedtime behavior of the day they were asked to recollect (‘yesterday’). Finally, at the end of the survey, participants were asked questions about their demographics.

**Table 1:** Overview of reported desires and resist attempts in relation to bedtime procrastination.

Desire	Total desires	Attempts to resist desires	Percentage of desires resulting in a resist attempt	BPS (*p*-value)	BP duration (*p*-value)
Eating	661	156	23.60%	0.17 (0.01)*	0.23 (>0.001)*
Media use	574	129	22.47%	0.07 (0.34)	0.09 (0.18)
Leisure	451	158	35.03%	0.09 (0.21)	0.13 (0.06)
Sleep	421	260	61.76%	0.22 (0.001)*	0.17 (0.014)*
Social contact	314	44	14.01%	0.15 (0.03)*	0.14 (0.04)*
Work	291	19	6.53%	-0.05 (0.48)	0.00 (0.99)
Coffee	264	40	15.15%	0.16 (0.02)*	-0.00 (0.98)
Hygiene	230	15	6.52%	0.08 (0.23)	0.05 (0.43)
Tobacco	160	30	18.75%	-0.01 (0.90)	-0.06 (0.42)
Non-alcoholic drinks	157	9	5.73%	0.02 (0.81)	-0.04 (0.53)
Sex	119	64	53.78%	0.09 (0.18)	0.01 (0.90)
Sports or Exercise	95	17	17.90%	-0.02 (0.77)	-0.01 (0.87)
Alcohol	72	30	41.67%	0.07 (0.30)	0.03 (0.71)
Spending	54	31	57.41%	0.14 (0.04)*	0.18 (0.01)*
Something else	28	5	17.86%	0.01 (0.94)	0.03 (0.67)

*The total number of experienced desires, sorted by frequency (“Total Desires”) as well as the number of resist attempts correlated with both measures of bedtime procrastination (BPS, “Bedtime Procrastination Scale”) using Spearman’s rank correlation method. The asterisk indicates p-value < 0.05 (please note that no corrections for multiple comparisons were carried out).*

To calculate the depletion index, we first calculated the sum of desires participants had tried to resist for each timeslot. So, if a participant indicated having experienced a desire for *coffee*, *leisure*, and *tobacco*, but only having tried to resist the latter two desires, the sum score for this timeslot would be 2. Thus, in line with previous research (Hofmann et al., 2012b), the depletion index was not based on the number of desires people experienced, only on the number of desires they had attempted to resist. Having a desire (e.g., longing for a cup of coffee in the morning) is not necessarily depleting; instead, trying to *resist* a desire (e.g., longing for a cigarette or cup of coffee, but trying not to give in to temptation because one is trying to live more healthily) requires self-control and is therefore thought to be depleting (Hofmann et al., 2012b).

After calculating the number of resist attempts per timeslot, we created a depletion index in which resist attempts that had taken place earlier in the day received less weight than resist attempts that had taken place later that day (cf. Hofmann et al., 2012b). Specifically, the most recent resist attempts received a weight of 6 (that is, their sum was multiplied by 6), resist attempts that had taken place a timeslot earlier received a weight of 5, etc., down to a weight of 1 for resist attempts that had taken place early in the morning. This way, attempting to resist a desire for *tobacco* and *leisure* in the morning had a relatively small effect on the total depletion index, whereas attempting to resist these desires just before going to bed had a relatively large effect on the depletion index. We calculated this *weighted depletion score* based on the assumption that, although depletion effects wear off over time, they carry a cumulative effect, such that resisting a desire for coffee or a smoke at 8 am has an effect on how depleted a person feels at 8 pm.

## Results

### Descriptive Results

As depicted in **Table 1**, there was a large difference in the number of resist attempts per desire. Eating, media use and leisure were the most commonly reported desires (reported 451–661 times), while sports, alcohol and spending where reported less frequently (reported 54–95 times). For certain desires, participants indicated attempting to resist them most of the time (e.g., when people experienced a desire for sleep or sex, they attempted to resist 61.76 and 53.78% of the time, respectively), while for other desires, participants hardly attempted to resist them (e.g., non-alcoholic drinks, work, and hygiene, were resisted 5.73, 6.53, and 6.52% of the time, respectively). The table also shows that sleep was reported quite frequently, and people often attempted to resist this desire (61.76% of the time). As such, this specific desire (which is plausibly related to bedtime procrastination) may affect the results, and we conducted our main analyses both with and without taking this specific desire into account.

### Main Analyses

To find out whether people who tried to resist more desires during the day were more likely to engage in bedtime procrastination, we first tested whether there was a correlation between the number of resist attempts (*weighted depletion index*) and bedtime procrastination (average score on the *BPS*). To deal with skewness, we calculated Spearman’s rank-order correlations instead of Pearson’s correlations for all analyses reported in the present study, unless noted otherwise. In line with our predictions, there was a significant correlation between *weighted depletion index* and the *BPS*, Spearman’s *r*(218) = 0.21, *p* = 0.001, indicating that people who reported that they had tried to resist many desires during the day were more likely to engage in bedtime procrastination. Put differently, participants who were depleted because they had attempted to resist many desires for a cigarette, cup of coffee, or other temptation throughout the day were more likely to engage in (what they considered to be) unwarranted delay of their bedtime.

As a second way of testing the relationship between bedtime procrastination and depletion, we calculated the correlation between the self-reported duration of the procrastination and the *weighted depletion index*. In line with our expectations, there was a significant correlation between these variables as well, Spearman’s *r*(218) = 0.17, *p* = 0.013, suggesting that people who reported that they had tried to resist many desires during the day showed a larger delay in bedtime that night. In other words, participants who were depleted because they had attempted to resist many desires throughout the day procrastinated more about going to bed.

Note that, of the day’s six “timeslots,” the duration of Timeslot 1 and Timeslot 6 (i.e., the time periods “between waking up and 9 am” and “between 9 pm and the time you went to bed”) depended on people’s individual sleep behavior and therefore varied across participants. This means that some participants would have had more time to experience and resist temptations than others in those timeslots (e.g., someone who woke up at 7 am would have had more time to experience and resist desires than someone who woke up at 8.45 am). To examine whether this affected our results, we also calculated the depletion index without taking these timeslots into account (so using Timeslots 2–5 only) and reran our correlation analyses. Notably, the pattern of results for both the BPS and Duration of Bedtime Procrastination remained the same.

Moreover, the pattern of results also remains the same when the depletion index is calculated without the desire for sleep. In line with our predictions, neither the BPS nor Duration of Bedtime Procrastination shows a significant correlation with the total number of experienced desires, with *r*s of 0.04 (*p* = 0.54) and 0.09 (*p* = 0.19), respectively.

## Discussion

The present study provides a first indication that resisting desires throughout the day may have a cumulative effect on people’s self-control in the evening. While the results only show a small to moderate effect size, the effect does appear to be robust, since varying the time periods included in calculating the depletion index, as well as including participants who met one or more of the exclusion criteria did not affect the pattern of results. This robustness is further corroborated by data from another survey study (unpublished) that also included the retrospective version of the Hofmann et al. (2012b) method and the retrospective BPS. With the data from this study (*N* = 270 in total; 31 participants were removed from the sample in the analyses because they suffered from a sleep disorder or worked night shifts), the BPS score and the weighted depletion score were calculated in the same way as in the main study and correlated using Spearman’s rank correlation. The resulting correlation followed the pattern of results for the present research [Spearman’s *r*(238) = 0.27, *p* < 0.001], again suggesting that, as we had hypothesized, people may indeed sometimes be “too depleted to turn in” after a taxing day.

Due to the nature of the study, however, it cannot be ascertained whether the participants in our study went to bed late because they truly lacked the self-regulatory resources to go to bed on time, or because of other factors. Because the study had a correlational design in which bedtime procrastination was measured retrospectively, it is possible that participants who engaged in bedtime procrastination recalled more resist attempts because they felt more tired. Using their current psychological state as a vantage point, participants may have overestimated the number of resist attempts they experienced the previous day.

Moreover, the findings from the present study can alternatively be explained by the idea that, after attempting to resist many desires throughout the day, participants are simply fatigued and find tasks around bedtime aversively effortful. Related possibilities are that bedtime procrastinators may be strategizing about how to allocate their effort (Inzlicht and Schmeichel, 2012; Berkman et al., 2017), or that they merely feel entitled to spend more time on the couch as a “reward” for trying to resist many temptations. In previous research, procrastination has been linked to short-term mood repair (Sirois and Pychyl, 2013), so it may also be that the bedtime procrastinators felt entitled to have a little more time for themselves after a demanding day (cf. *licensing effects*; De Witt Huberts et al., 2014). Thus, in some cases, the effect of resource depletion on procrastination could be moderated, mediated, or replaced by participants’ (unwarranted) belief that they need or deserve extra time to “unwind” and relax before turning in.

As an additional point, it is worth noting the possibility of a bidirectional effect, such that people may have more self-regulatory resources as a result of not regularly engaging in bedtime procrastination and thus being less chronically depleted by sleep deprivation. For these people, potential conflicts between long-term goals and desires may be handled effortlessly and automatically (Gillebaart and De Ridder, 2015), so that these people report experiencing fewer desires as temptations. Conversely, people who get less sleep are likely to have more difficulty resisting desires (e.g., Christian and Ellis, 2011).

Alternative explanations such as these would have to be ruled out before a stronger causal link between resisting desires throughout the day and bedtime procrastination can be established. Future research could utilize a different methodological approach to counteract this problem. For example, qualitative methods such as conducting semi-structured interviews with bedtime procrastinators could be utilized to gain more insight into the psychological mechanisms of bedtime procrastination. Furthermore, future studies could utilize experience sampling methods, in which participants are asked several times a day to report if they experience desires that they are trying to resist (cf. the study by Hofmann et al., 2012b). The use of experience sampling would make it possible to ask participants additional questions about the emotions and cognitions they experience while delaying their bedtime, and about the reasons they have for their delay (e.g., “I’m too tired” versus “I deserve some time for myself”).

Dynamically prompting participants to answer questions while they are engaging in bedtime procrastination (e.g., by sending them questions after their intended bedtime), would help to better understand what happens at the exact moments that people are putting off their bedtime. Such research could also be expanded to exclude possible effects of moments during the days when participants’ self-regulatory resources are “restored” by various factors (cf. Masicampo et al., 2014), including satisfying (rather than resisting) desires. It would also introduce the possibility of examining whether the activities engaged in during bedtime procrastination are experienced as less pleasurable (guilt-tainted “spoiled pleasures”) or more pleasurable (indulgence in “forbidden fruit”) when participants think of themselves as procrastinating (Hofmann et al., 2013; see Anderson, 2016 on the related *mens rea* requirement for procrastination).

As it stands, the preliminary findings from the present research do provide additional evidence in support of the idea that, in ways similar to failing to maintain a healthy diet, quit smoking, or get sufficient exercise (De Ridder et al., 2012), bedtime procrastination can be a regarded as a form of self-regulatory failure. However, we suspect that cumulative effects of resisting desires may be even more pronounced for bedtime procrastination than for many other self-regulation conflicts. For although most self-regulation conflicts occur at different moments distributed throughout the day, the conflict between the desire to indulge in a bedtime-delaying activity and the desire to get sufficient sleep (by sticking to one’s bedtime) always takes place at the point in one’s day when cumulative depletion tends to be at its peak. For this reason, understanding the effects of cumulative depletion may be particularly important to develop effective interventions to diminish bedtime procrastination. Given the evidence that self-regulation failure is correlated with sleep insufficiency in the general population, strategies that have proven effective in combatting self-regulatory failure in other areas of health behavior, such as monitoring goal progress (Harkin et al., 2016), may be fruitful in combatting sleep insufficiency. However, the present study suggests that a more comprehensive coaching and persuasive intervention strategy might be necessary, as people may be running low on self-regulatory resources at night. To the extent to which these preliminary findings are borne out by future research, there are reasons to design interventions aimed at reducing bedtime procrastination in such a way as to avoid overburdening people at the end of a long, hard day. In this regard, given the evident importance of understanding the state of one’s self-regulatory resources in navigating (away from) situations of temptation, it could prove useful to find reliable ways of anticipating the extent to which, on a given evening, one is likely to be particularly susceptible to bedtime procrastination.

There may be evenings on which it is a bad idea to start watching a favorite TV series close to bedtime, and there may be evenings on which it is no problem at all. Knowing which is which can be tricky, particularly if one’s capacity to resist self-indulgent rationalization declines with self-regulatory resources at the end of the day. Whether or not it will ever be possible to develop “ubiquitous technology” (such as wearables) to monitor the number of resist attempts one has engaged in during a day, the findings of the present study lend credence to the notion that successful self-regulation and self-management may well be improved by finding ways of reliably tracking the extent to which one’s end-of-the-day resources have been depleted. More research would clearly be needed to assess the viability, desirability, and ethics of developing such techniques. What the findings of our study provide is an initial indication that reliable information about depletion levels may well be a key component of effective strategies for predicting and reducing bedtime procrastination and other instances of late-night self-regulation failure.

## Ethics Statement

This study was carried out in accordance with the research protocol of Utrecht University’s Social Science Ethical Committee. All subjects gave written informed consent in accordance with the Declaration of Helsinki.

## Author Contributions

All authors have made substantial contributions to the conception of the work, the interpretation of the findings, and the drafting of the manuscript. All authors agreed to be accountable for the content of the work and have given their approval for publication.

## Conflict of Interest Statement

The authors declare that this study was carried out with the support of grant #12013 from the Technology Foundation STW’s “Healthy Lifestyle Solutions” Partnership program, which is jointly funded by the Netherlands Initiative on Brain and Cognition (NWO) and Philips Research. The funders were not involved in the design of the study, nor in the collection, analysis, or interpretation of the data.

## Footnotes

1 A randomized controlled trial in this direction has been performed in which participants were asked to keep a sleep diary and were provided with two floor lamps to place in their living room and bedroom. In the experimental condition, the lights in these floor lamps would briefly flash and subsequently change hue at the participant’s intended bedtime. Participants in this condition were asked to couple this environmental cue to an implementation intention targeted at going to bed (e.g., “If the light changes hue, I will go upstairs to brush my teeth and go to bed”). The collected data from this experiment support the idea that such behavioral interventions can be effective at reducing people’s bedtime procrastination. The experimental setup and results are presented in a manuscript that is currently under review.

2 Exclusion criteria were determined *a priori*. The pattern of significant and non-significant results does not differ regardless of whether these people were retained in the sample or not.
